# Food preference and behavioural choice across the eating disorder and body weight spectrum

**DOI:** 10.1186/s40337-026-01547-4

**Published:** 2026-02-14

**Authors:** K. N. Eichin, A.-K. Arend, J. Reichenberger, U. Voderholzer, J. Blechert

**Affiliations:** 1 Department of Psychology and Centre for Cognitive Neuroscience, University of Salzburg, Hellbrunner Straße 34, Salzburg, 5020 Austria; 2 Institute of Psychology, Johannes Kepler University Linz, Altenberger Str. 69, Linz, 4040 Austria; 3 Schoen Clinic Roseneck, Am Roseneck 6, 83209 Prien am Chiemsee, Germany; 4 LMU Clinic Munich, Nußbaumstraße 7, 80336 München, Germany

**Keywords:** Eating disorder spectrum, Anorexia nervosa, Bulimia nervosa, Binge eating disorder, Overweight, Obesity, Food choice, Food preferences, Choice processes, Reaction times

## Abstract

**Background:**

Eating disorders (EDs) such as Anorexia Nervosa (AN), Bulimia Nervosa (BN), and Binge-Eating Disorder (BED) share overlapping symptoms, risk factors, and maintenance mechanisms, yet differ in their behavioural manifestations. This study aimed to investigate binary food choice behaviour across EDs and weight groups, including healthy controls with normal weight, overweight and obesity in a controlled laboratory setting.

**Methods:**

*N* = 189 individuals with AN (*n* = 40), BN (*n* = 29), BED (*n* = 24) as well as controls without eating disorders with normal weight (HC-NW, *n* = 57), overweight (HC-OW, *n* = 18) and obesity (HC-OB, *n* = 21) made 153 binary food choices while decision times were recorded. Participants also rated foods on liking and calorie content. The number of calories in chosen foods, the relationship between calorie content and liking ratings and decision times were analysed. We further explored the roles of eating styles, BMI and ED symptoms in food choice.

**Results:**

Individuals with AN chose less calories, liked lower-calorie foods more and made faster decisions, especially for lower-calorie options compared to HC-NW. The other groups did not differ from HC-NW in food choice or liking, but BN - and to a lesser degree HC-OB and HC-OW - made faster food choices. External eating positively predicted higher-calorie choices.

**Conclusion:**

This study underscores the distinctiveness of AN in food choice behaviour and the need for transdiagnostic approaches to understand shared and unique mechanisms across EDs and weight groups. Future research should explore longitudinal changes in food choice processes and integrate contextual and emotional factors to better capture the complexity of eating behaviours.

**Supplementary Information:**

The online version contains supplementary material available at 10.1186/s40337-026-01547-4.

## Abstract (plain language summary)

This study looked at how people with different eating disorders (Anorexia Nervosa, Bulimia Nervosa, and Binge-Eating Disorder) and different body weights (normal weight, overweight, and obesity) make food choices. Participants completed a computer-based task in which they chose between pairs of foods, rated how much they liked them, and estimated their calorie content. It was also measured how quickly they made the decisions. The results showed that people with Anorexia Nervosa chose lower-calorie foods more often, liked them more, and made faster decisions when they chose them compared with people of normal weight who did not have eating disorders. Other groups, including those with Bulimia Nervosa, overweight, or obesity, did not differ in the foods they liked but made faster decisions overall. People who tended to eat in response to external cues (e.g., seeing food) were more likely to choose higher-calorie options. The study highlights how food choices are different for people with Anorexia Nervosa and suggests that eating disorders share some similarities but also have unique traits. We recommend studying how food choices change over time and considering emotional and situational factors to better understand eating behaviours.

**Supplementary Information:**

The online version contains supplementary material available at 10.1186/s40337-026-01547-4.

## Background

Eating disorders do not only overlap in core symptoms such as fasting (in Anorexia Nervosa (AN)) and Bulimia Nervosa (BN), binging (in the binge-purge type of AN, BN and Binge-Eating Disorder (BED)) and purging (in the binge-purge type of AN and BN) but also share risk factors such as negative affectivity and problematic parenting [1] and maintenance factors as suggested by transdiagnostic theories. These include low self-esteem and interpersonal difficulties [2]. While individuals with overweight and obesity do not show pathological eating behaviours such as extreme dieting or binge eating, they share genetic risk variants with BN and BED [3] and are more likely to diet in an attempt to lose weight [4]. Although it is unclear whether dieting behaviour in itself constitutes a risk for developing an eating disorder [5], it is strongly associated with food concern [6] and therefore a more conflicted stance towards food. Importantly, these shared factors might affect *food choice* and thereby composition and quantity of food intake. Thus, to contribute to future transdiagnostic theory building and intervention, the present study compared individuals with eating disorders (AN, BN and BED) as well as individuals with overweight (HC-OW) and obesity (HC-OB) with healthy weight controls on food choice and food liking in a controlled laboratory environment. But why is food choice so central?

In today’s affluent societies and food-rich environments, individuals make hundreds of eating-related decisions every day [7], often involving resisting current intake. From an evolutionary perspective, the primary driver of eating was homeostatic need (or ‘hunger’), yet modern human food choice is more complex: It is influenced by hedonic reasons such as taste, convenience, habits, mood, ethical reasons, health or weight considerations, among others [8]. The multitude of determinants gives rise to *decision-making conflicts*, such as when health considerations and taste preferences compete (“tasty but unhealthy”). While such conflicted decisions can be seen in many healthy individuals at times, they are arguably more frequent, intense, and debilitating in individuals with eating disorders. For individuals with BED and BN, deliberate food choice can turn into impulsive, uncontrolled binge eating. By contrast, in those with the restrictive type of AN, food choice is often the choice *not* to eat, or to reduce the calorie density of foods to reach or maintain a very low body weight. Individuals with BN additionally seek to compensate for their uncontrolled eating with compensatory measures, such as vomiting or fasting [9]. This sets up potential conflicts when it comes to food choice: the desire to eat palatable foods competes with weight concerns. Some of these conflicts might be shared by groups with higher weight, i.e., obesity (body mass index (BMI) > 30 kg/m2) or the subclinical condition overweight (BMI 25–30 kg/m²).

As actual food choices can be difficult to investigate in individuals with eating disorders, especially during inpatient treatment (frequent in AN), hypothetical food choice tasks can be used as a form of symptom-provocation (cf. 10). They do not only have clear relevance to everyday life shopping and eating behaviour but also provide valuable insights into reward processing [11], decision making and self-control [12] while having high reliability [13] and acceptable validity for actual eating behaviour [14, 15]. Food choice tasks also offer the advantage of analysing decision times as an additional measure of *how* food choices are made. Dual-process theories suggest that cognitive processes take place in two different manners: one that is fast and automatic and one that is slower and deliberate (see e.g., [16]). How food choices are made (fast vs. deliberate) might differ between groups, with individuals with binge symptomatology possibly making faster choices and individuals with AN being more on the deliberate side.

Despite these advantages, behavioural food choice studies are surprisingly rare in groups with higher weight and in eating disorders. In laboratory food choice studies, individuals with BED both liked and chose higher-calorie foods more often than healthy controls with similar weight [17]. Individuals with BN chose higher-calorie foods slightly more frequently in one study [18] but chose fewer high-fat foods in another study [19]. Differences in choice outcomes may be influenced by emotional state: while both BN and HC showed a choice bias for high-fat foods in a negative-affective state, this tendency was stronger in BN [20]. Individuals with AN chose high-fat foods less often [14, 21] and were more influenced by the healthiness of foods compared with healthy controls [22]. The proportion of high-fat choices increases during treatment [23]. Although some studies report alterations regarding reward-related decision-making in overweight and obesity, evidence is mixed, and most studies focus on non-food decisions [24]. Thus, to our knowledge, no study has directly compared food choice behaviour in AN, BN and BED and higher-weight groups to normal weight controls (HC-NW).

The present study reanalysed partially published laboratory decision-making data [15, 17, 18] to allow direct comparisons between the different eating disorder and weight groups and HC-NW. A total of 189 individuals made 153 computer-based binary food choices, rated 18 foods for calorie content and liking and completed questionnaires. We first examined whether groups deviated from HC-NW in the number of calories they chose (i.e., the calories in foods selected during binary choice). Since previous results from an overlapping sample suggest that individuals with higher BMI without an eating disorder are more likely to choose lower-calorie foods compared with individuals with moderately elevated or normal BMI [17], we expected the following ranking of calories chosen: AN < OB < OW < NW < BN/BED (*calorie choice hypothesis*). Second, we examined food preferences by investigating the within-individual relationship between liking and calorie ratings across the 18 foods (*calorie-liking hypothesis)*. Here, we expected the lowest liking for higher-calorie foods in AN [18]. In addition, our previous findings suggest higher liking for higher-calorie foods in BED compared to HC-NW. It was unclear whether BN, HC-OW and HC-OB would differ from HC-NW. Third, we explored whether decision times for food choices differed between groups as a function of the calorie-content of chosen foods (*decision time analysis).* In other words, are choices for higher-calorie food faster or slower than choices for lower-calorie foods?

## Methods

### Sample

All participants were female. Participants underwent two clinical interviews (Eating Disorder Examination and the Structured Clinical Interview for DSM-IV;,25,26) updated to DSM-5^1^. Trained master’s students, PhD students and postdoctoral researchers conducted the interviews. The sample initially comprised *n* = 40 individuals with AN, *n* = 32 individuals with BN and *n* = 24 individuals with BED. The control group consisted of *n* = 108 individuals without eating disorders. Individuals with AN and BN were recruited at a German treatment centre for eating disorders (Schoen Klinik Roseneck, Prien am Chiemsee), where they were receiving inpatient care. Individuals with BED^2^ and the HC groups were tested at the University of Salzburg. Some HC participants were excluded because of eating disorder symptoms (*n* = 3), underweight (*n* = 5), current substance abuse (*n* = 1) or fewer than 45 valid choice trials (*n* = 3). In the group with BN *n* = 3 were excluded because of atypical BN (*n* = 1), not following task instructions (*n* = 1), and strong food disgust (*n* = 1). The final sample of *N* = 189 consisted of *n* = 96 HC (*n* = 57 normal weight, *n* = 18 overweight and *n* = 21 with obesity), *n* = 40 with AN (of which *n* = 31 AN-res, *n* = 9 AN-bp), *n* = 29 with BN and *n* = 24 with BED. Regarding nationality, *n* = 104 participants were German, *n* = 81 participants were Austrian, *n* = 3 reported another nationality and *n* = 1 did not answer the question. We expect these groups to be similar in their cultural food background. See Table 1 for sample characteristics. Groups differed on age and BMI, as expected given the different eating pathologies and their typical onset. Because the groups with AN and BN did not follow the pre-session treatment protocol owing to treatment regulations, they also differed in hunger. Groups did not differ in years of education.

**Table 1:** Sample characteristics

	HC-NW (*n* = 57)	HC-OW (*n* = 18)	HC-OB (*n* = 21)	AN (*n* = 40)	BN (*n* = 29)	BED (*n* = 24)	ANOVA group differences
Age	22.6 (6.04)	26.11 (5.74)	32.76 (8.17)	23.1 (5.28)	22.62 (5.73)	34.13 (10.65)	*P* <.001
Years of education	14.66 (2.56)	15.81 (3.02)	14.3 (3.47)	14.89 (2.96)	13.62 (2.2)	15.54 (4.89)	*P* =.17
BMI	21.96 (1.78)	27.54 (1.61)	37.58 (6.19)	15.50 (1.78)	23.48 (4.57)	30.32 (6.03)	*P* <.001
Hunger	4.18 (1.93)	3.94 (1.51)	4.14 (2.24)	1.98 (1.33)	2.52 (1.72)	4.38 (1.95)	*P* <.001

*AN* Anorexia Nervosa, *BN* Bulimia Nervosa, *HC-OW* Healthy controls with overweight, *HC-OB* Healthy controls with obesity, *BED* Binge-Eating Disorder. The AN sample consisted of *n* = 31 individuals with the restricting type of AN and *n* = 9 individuals with the binge-purge type of AN. Significant differences in hunger reflect differences between the groups that took part in the lunch standardisation (all HC and BED) and those in inpatient treatment who followed meal plans as part of treatment (AN and BN)

### Procedure

**Fig. 1:**
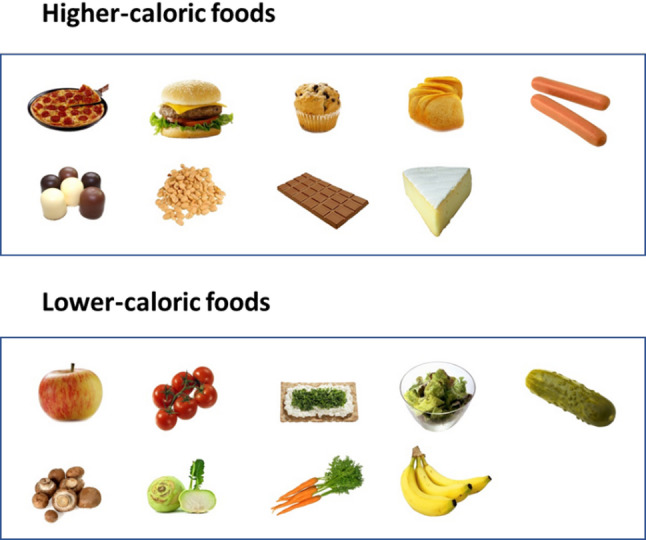
Overview of food images. Images were retrieved from the food-pics database [29]. Image numbers are listed in the supplement

**Fig. 2:**
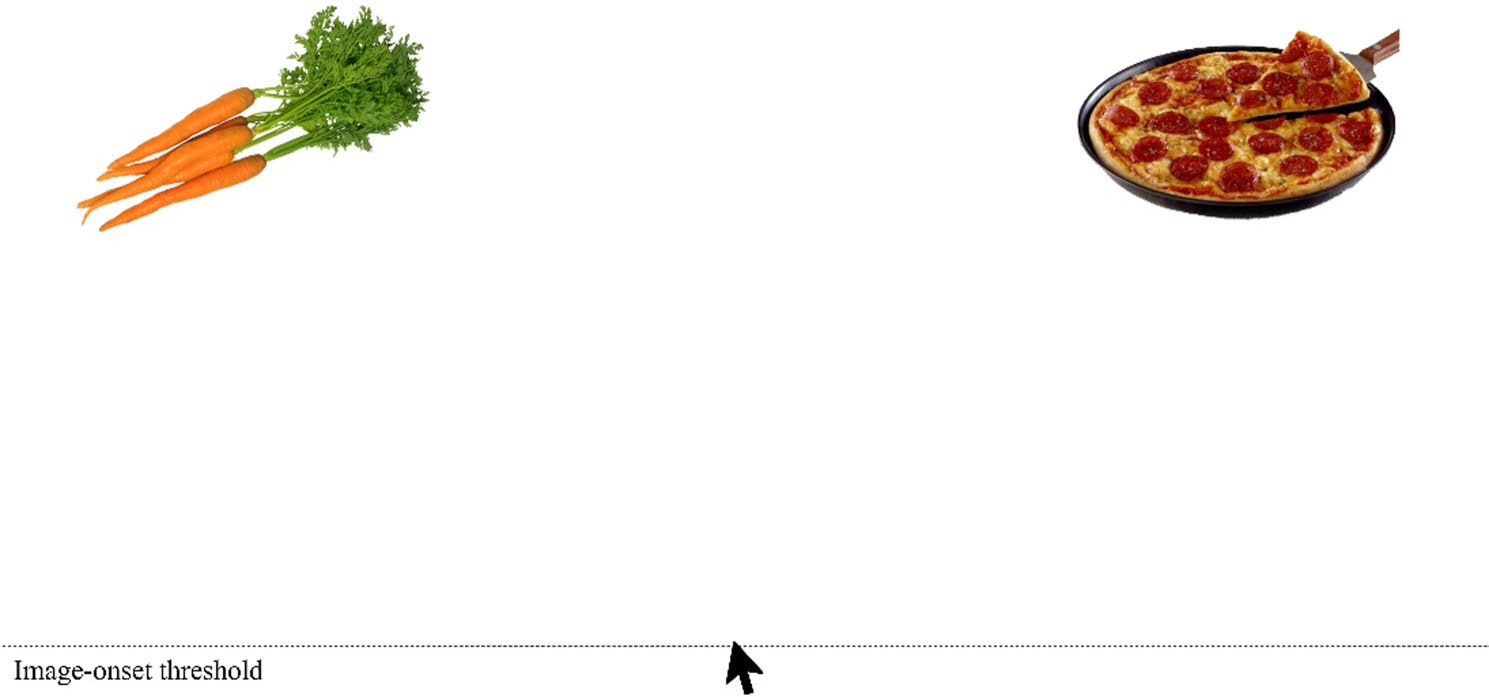
Choice trial example. The image shows an example trial from the choice task. Images appeared when the mouse crossed an invisible threshold (shown in the figure for illustrative purposes)

Prior to the laboratory part of the experiment, participants completed online questionnaires (Dutch Eating Behaviour Questionnaire (DEBQ; [27]) and Eating Disorder Examination Questionnaire (EDE-Q; [28])). To partially standardise homeostatic state, all participants except for those with AN and BN (who followed their therapeutic meal plans) were asked to consume one of five suggested lunch options of approximately 550 kcal, three hours before the start of the experiment and to refrain from eating thereafter. Although energy needs and eating habits differ between individuals, we expected this strategy to limit variability in hunger levels between participants. Participants with AN and BN followed their in-treatment meal plans, so lunch standardisation was not possible in these groups. We attempted to keep the time between lunch and experiment similar to the control group, but scheduling led to variation of approximately 2–3.5 h. In the laboratory, participants made 153 binary food choices between food pairs composed from all unique combinations of 18 different higher^3^- and lower-calorie^4^ foods retrieved from the food-pics database [29]. For on overview of the food images, see Fig. 1 (image numbers are listed in the supplement). For an example of the choice trial, see Fig. 2.

The foods were selected to represent the typical local food environment, varying in healthiness and calorie density. We focused on total calories rather than fat (see e.g., [23, 30]), to present participants with a wider range of foods. We also expected that total calories, not just calories from fat, would be relevant to individuals with weight/shape-concerns. They were asked to choose the food that they would like to eat and were told that they would receive their five most frequently chosen foods after the task. There were no further instructions regarding the portion size or any expectancy about how much to eat of it. Participants were instructed to click a start button, then continuously move the mouse upwards. When an invisible threshold was crossed, food images were presented. Decision times were recorded to the nearest millisecond. Images were presented using E-Prime [31]. After the food-choice task, participants rated all foods on liking and calorie content (as well as health and desire to eat, which were not used for this manuscript) on a visual analogue scale ranging from 0 (not at all) to 100 (completely). Liking was explained to participants as general liking of a food, complementary to desire to eat (‘wanting’) the food at that moment. We decided to use liking and desire to eat instead of ‘tasty’ as in some previous studies (see e.g., [32, 33]) because tasty might tap both into the liking and wanting component.

### Data analysis

Trials that exceeded a maximum duration of 4 s were excluded. For the *calorie-choice analysis* a Bayesian multi-level model (mlm) with chosen calories as outcome variable and group as predictor was fitted in brms [34] using a negative binomial model. The model included a random intercept for each participant. For the *calorie-liking hypothesis*, the relationship between liking and calorie content was modelled using a Bayesian mlm with liking rating as outcome variable and calorie rating, group and their interaction as predictors. Because of its distribution, liking was transformed to use a model of the beta family. For the *decision time hypothesis*, a Bayesian linear model with a shifted log-normal distribution was used to predict decision times by chosen calories and diagnostic group was used. In all analyses, group was coded as a dummy-variable with HC-NW as comparison group.

Given the similarity of all non-AN groups in food choice and liking, we further explored which characteristics other than group influenced food choice. Accordingly, we conducted an exploratory analysis of predictors of choice including only the non-AN groups. A Bayesian MLM with chosen calories as the outcome and BMI, EDE-Q, DEBQ-emotion and DEBQ-external as predictors was fitted. There were no divergent transitions and *Rhat* < 1.01 for all relevant model parameters.

## Results

### Calorie-choice hypothesis

**Fig. 3:**
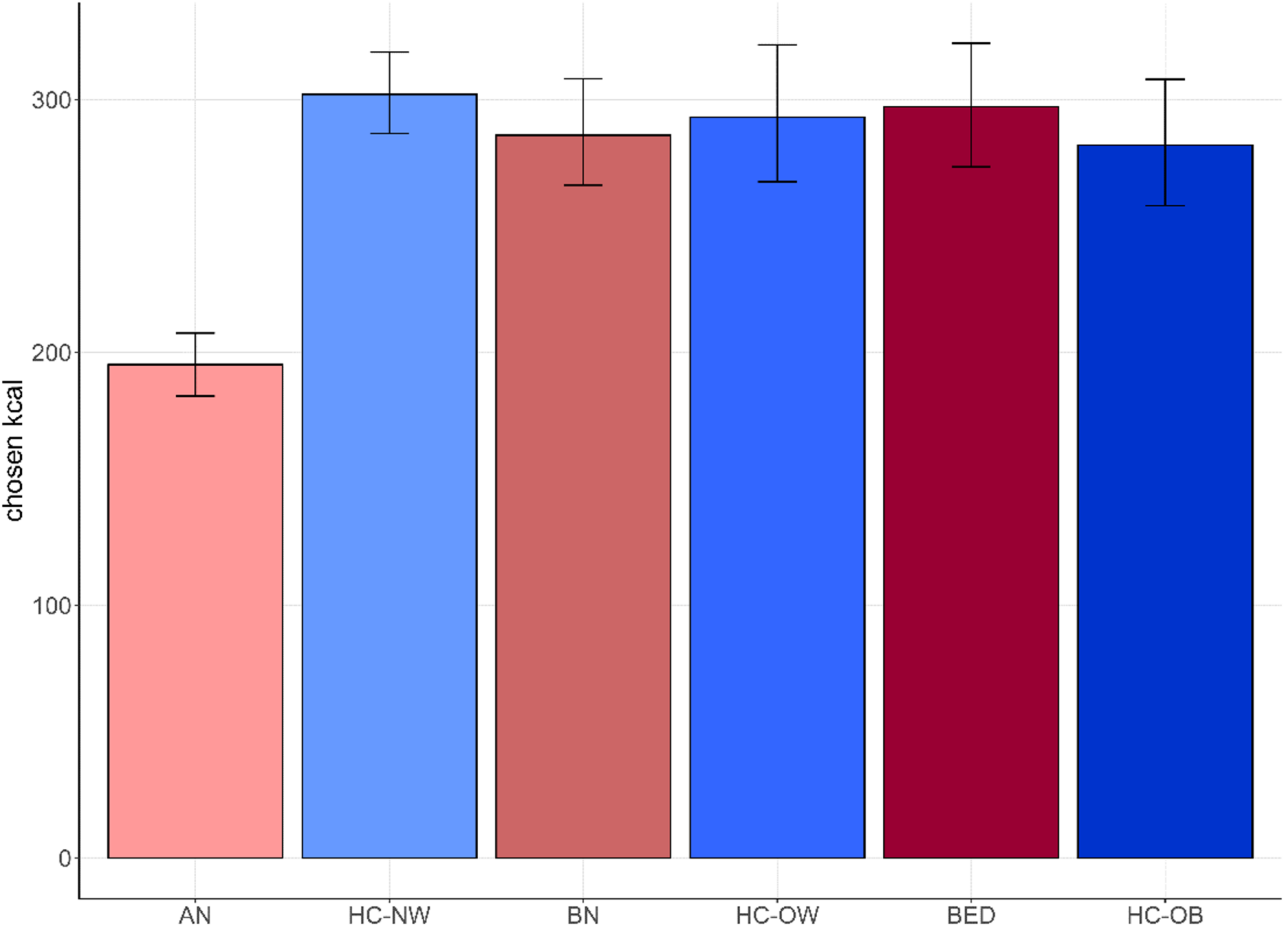
Calorie-choice analysis. Results of the Bayesian hierarchical model predicting the number of calories chosen by group. Groups are ordered by BMI, with the lowest BMI on the left. Groups with eating disorders are displayed in red shades; the groups without eating disorders are displayed in blue shades. Error bars display the 95% credible interval. *Kcal* kilocalories, *AN* Anorexia Nervosa, *HC-NW* Healthy controls with normal weight, *BN* Bulimia Nervosa, *HC-OW* Healthy controls with overweight, *BED* Binge-Eating Disorder, *HC-OB* Healthy controls with obesity

Contrary to expectations, the Bayesian multi-level model for calorie choice showed that only AN differed from HC-NW by choosing fewer calories: (AN: *b* = − 0.44, CI: [−0.52; −0.35]; see Fig. 3; Table 2). The other groups HC-OW and HC-OB (blue in Fig. 3) as well as BED and BN (red in Fig. 3) did not differ from HC-NW (blue).

**Table 2:** Calorie-Choice analysis

	Estimate	std. Error	CI (95%)
Intercept	5.71	0.03	5.66; 5.76
AN	**−0.44**	0.04	**−0.52; −0.35**
BN	−0.05	0.05	−0.14; 0.03
HC-OW	−0.03	0.05	−0.13; 0.08
HC-OB	−0.07	0.05	−0.18; 0.04
BED	−0.02	0.05	−0.11; 0.08

Results of the Bayesian multi-level model for the number of chosen calories predicted by group. HC-NW is the reference category for the dummy-coded group variable against which the other groups are compared. Bold indicates that the credible interval excludes 0. *AN* Anorexia nervosa, *BN* Bulimia Nervosa, *HC-OW* Healthy controls with overweight, *HC-OB* Healthy controls with obesity, *BED* Binge-Eating Disorder

### Calorie-liking hypothesis

**Fig. 4:**
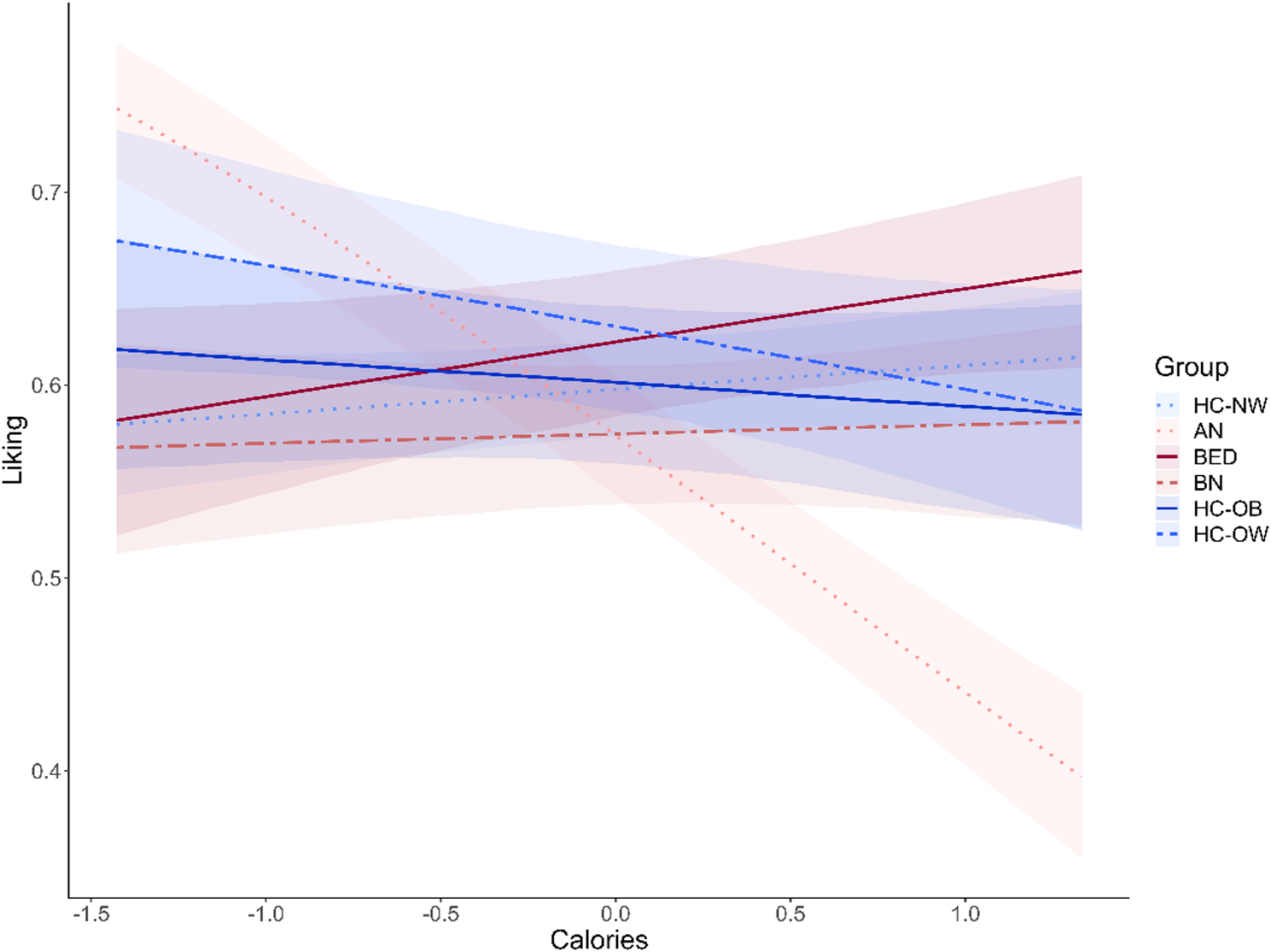
Calorie-liking relationship. Results of the Bayesian multi-level model for the relationship between calorie ratings and liking ratings. Groups with eating disorders are displayed in red shades; the control groups are displayed in blue shades. Error bands display the 95% credible intervals. *AN* Anorexia Nervosa, *HC-NW* Healthy controls with normal weight, *BN* Bulimia Nervosa, *HC-OW* Healthy Controls with overweight, *BED* Binge-Eating Disorder, *HC-OB* Healthy controls with obesity

For the relationship between liking and calorie content, again, AN (*b* = − 0.59, CI: [−0.71; −0.46]) differed significantly from HC-NW. Their liking-calorie relationship was more negative, i.e., they liked lower-calorie foods more than higher-calorie foods. To a lesser degree, this was also the case in HC-OW (*b* = − 0.19, CI: [−0.35; −0.02]). In contrast, individuals with BED showed slightly higher liking for higher-calorie foods (*b* = 0.07, CI: [−0.08; 0.21]), but this was not significant. Individuals with BN and HC-OB did not differ significantly from HC-NW (Fig. 4; Table 3). For additional group-wise correlations between healthiness and liking, see supplement.

**Table 3:** Calorie-liking analysis

	Estimate	std. Error	CI (95%)
Intercept	0.40	0.05	0.30; 0.50
z_Calories	0.05	0.04	−0.03; 0.13
AN	−0.10	0.08	−0.26; 0.06
BN	−0.10	0.09	−0.27; 0.08
HC-OW	0.14	0.11	−0.08; 0.35
HC-OB	0.01	0.10	−0.19; 0.21
BED	0.10	0.10	−0.09; 0.29
Calories*AN	**−0.59**	0.06	-**0.71; −0.46**
Calories*BN	−0.03	0.07	−0.17; 0.11
Calories*HC-OW	**−0.19**	0.08	**−0.35; −0.02**
Calories*HC-OB	−0.10	0.08	−0.25; 0.05
Calories*BED	0.07	0.07	−0.08.; 0.21

Results of the Bayesian multi-level model predicting liking from calorie content and group. HC-NW is the reference category for the dummy-coded group variable against which the other groups are compared. Bold indicates that the credible interval excludes 0. *AN* Anorexia nervosa, *BN* Bulimia Nervosa, *HC-OW* Healthy controls with overweight, *HC-OB* Healthy controls with obesity, *BED* Binge-Eating Disorder

### Decision time analysis

**Fig. 5:**
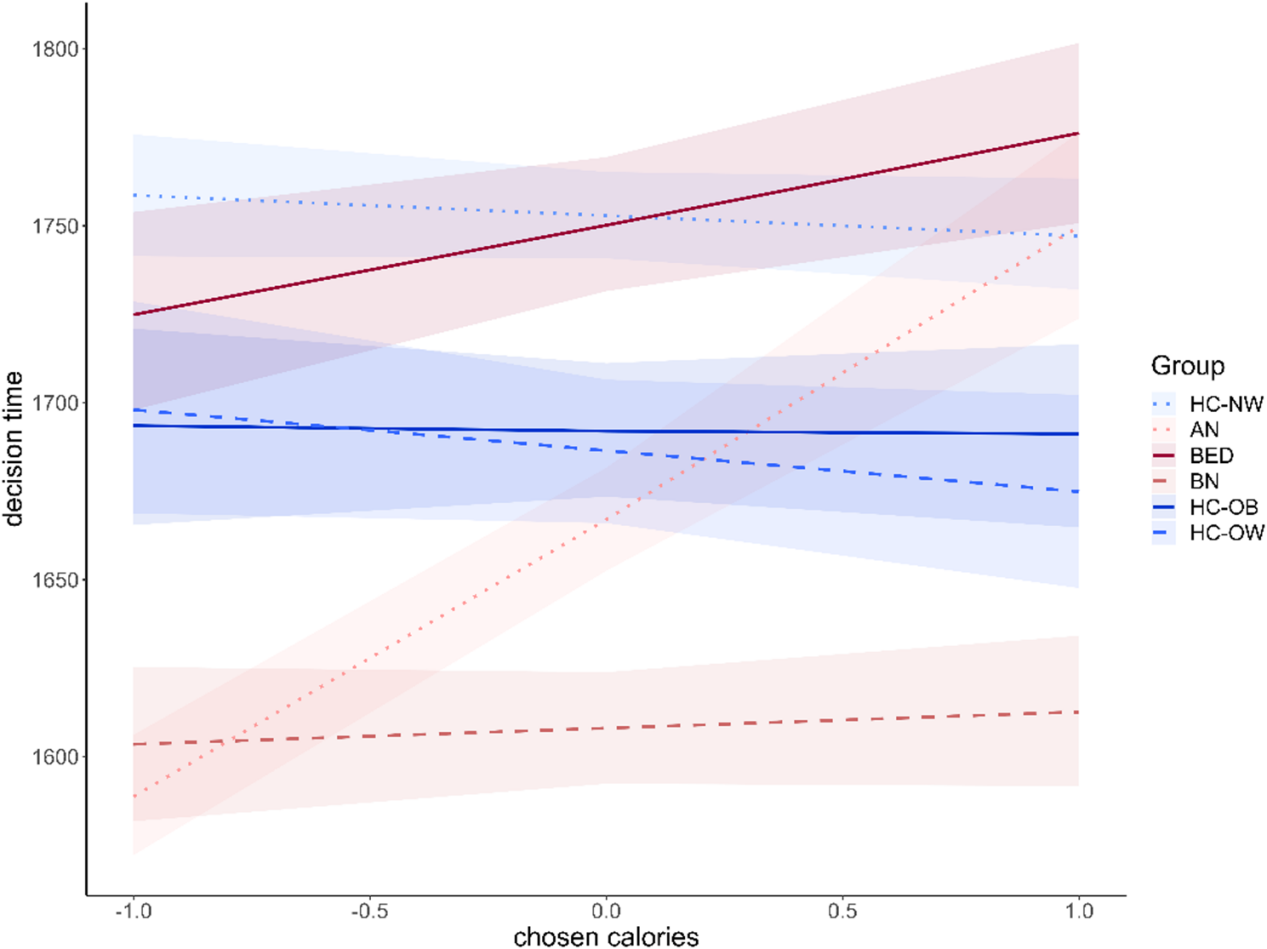
Decision time analysis. Results of the Bayesian linear model for the analysis of decision times dependent on group and calories of the chosen food. Groups with eating disorders are displayed in red shades; the control groups are displayed in blue shades. Error bars display the 95% credible intervals. *AN* Anorexia Nervosa, *HC-NW* Healthy controls with normal weigh, *BN* Bulimia Nervosa, *HC-OW* Healthy Controls with overweight, *BED* Binge-Eating Disorder, *HC-OB* Healthy controls with obesity

Regarding general group differences in decision time (main effects), individuals with BN (*b* = − 0.11, CI: [−0.12; −0.09]) were generally faster in their food choices than HC-NW (see Fig. 5; Table 4). To a lesser degree, this was also the case for individuals with AN (*b* = − 0.06, CI: [−0.08; −0.05]) and the control groups with overweight (*b* = − 0.05, CI: [−0.07; −0.03]) and obesity (*b* = − 0.04, CI: [−0.06; −0.03]). Beyond these general decision time differences, decision time was moderated by the calorie content of chosen foods in some groups. In AN (*b* = 0.07, CI: [0.05; 0.08]) choices for lower-calorie options were faster than choices for high-calorie options. To a lesser degree, this was also the case in BED (*b* = 0.02, CI: [0.01; 0.04]), but the effect was very small.

**Table 4:** Decision time analysis

	Estimate	std. Error	CI (95%)
Intercept	7.16	0.01	7.14; 7.18
Kcal chosen	−0.00	0.00	−0.01; 0.00
AN	**−0.06**	0.01	**−0.08; −0.05**
BN	**−0.11**	0.01	**−0.12; −0.09**
HC-OW	**−0.05**	0.01	**−0.07; −0.03**
HC-OB	**−0.04**	0.01	**−0.06; −0.03**
BED	−0.00	0.01	−0.02; 0.01
AN*kcal chosen	**0.07**	0.01	**0.05; 0.08**
BN*kcal chosen	0.01	0.01	−0.01; 0.02
HC-OW*kcal chosen	−0.00	0.01	−0.02; 0.01
HC-OB*kcal chosen	0.00	0.01	−0.01; 0.02
BED*kcal chosen	**0.02**	0.01	**0.01; 0.04**

Results of the Bayesian multi-level model predicting liking from calorie content and group. HC-NW is the reference category for the dummy-coded group variable against which the other groups are compared. Bold indicates that the credible interval excludes 0. *AN* Anorexia nervosa, *BN* Bulimia Nervosa, *HC-OW* Healthy controls with overweight, *HC-OB* Healthy controls with obesity, *BED* Binge-Eating Disorder

### Exploratory predictors of choice analysis

Since all non-AN groups (BN, BED, HCs) were similar in calories chosen, we explored whether variables other than diagnostic group better predicted food choice in these groups. We therefore explored the role of eating disorder symptoms in a dimensional way using the EDEQ [28]. Additionally, prior research suggests that emotional eating might be influential, but also BMI [35] and external eating [36]. Thus, we predicted choice from BMI, EDE-Q, DEBQ-emotional and DEBQ-external eating (including interactions of BMI with the questionnaire scores). This analysis identified DEBQ-external as the only significant predictor of food choice (*b* = 0.06, CI: [0.03; 0.09], see also Table 5). Higher external eating scores were associated with choosing more calories.

**Table 5:** Exploratory predictors of choice analysis

	Estimate	std. Error	CI (95%)
Intercept	5.67	0.02	5.64; 5.7
BMI	−0.01	0.02	−0.04; 0.02
DEBQ-Emotion	0.01	0.02	−0.03; 0.05
DEBQ-External	0.06	0.02	**0.03; 0.09**
EDE-Q	0.00	0.02	−0.04; 0.05
BMI*DEBQ-emotion	0.03	0.03	−0.02; 0.09
BMI*DEBQ-external	−0.02	0.02	−0.06; 0.01
BMI*EDE-Q	0.03	0.03	−0.02; 0.08

Results of the Bayesian multi-level model for the number of chosen calories. Bold indicates that the credible interval excludes 0. *DEBQ* Dutch eating behaviour questionnaire, *EDE-Q* Eating disorder examination questionnaire, *BMI* Body Mass Index

## Discussion

This study was the first to compare all major eating disorder and weight groups with normal weight controls on laboratory food choice. The results are discussed as follows.

The only group clearly distinct from HC-NW was AN. They chose significantly fewer calories than HC-NW, liked lower-calorie foods better and were faster to choose them than high-calorie foods. This increased liking of lower-calorie foods might help maintain their deeply internalized avoidance of high-calorie foods [37]. This is in line with Foerde et al. [38] suggesting distinct neural mechanisms underlying food choice in AN. Consistent with the other measures, the AN group showed faster decision times than HC-NW, especially when choosing lower-calorie foods as compared to higher-calorie foods. This might reflect that the latter choices were more difficult for them. Compared with results of other indirect measures, this is in line with the negative implicit bias for higher- but not lower-calorie foods AN showed in the affect misattribution procedure [39].

As for the non-AN groups, surprisingly, neither individuals with BN nor individuals with BED differed significantly from HC-NW in the number of *chosen calories* or *calorie liking*. There are several possible explanations for this. First, we did not systematically induce negative emotions or stress immediately before food choice, which might have created conditions similar to antecedents of binge eating (see e.g., [40]). Unlike AN, who follow strict eating-rules, both BN and BED still consume a broad spectrum of foods, which might be reflected in their choice behaviour and calorie liking. Second, we used the same images for all participants, which improves comparability but might have had different effects than using personal binge-foods [41]. Third, food choices might also have been influenced by social desirability in the laboratory setting. Fourth, although individuals who suffer from binge eating clearly differ in their food selection during binge-episodes, this might not be reflected in their choices and preferences between episodes. Similarly, in a dietary recall study [42], individuals with BEDs calorie consumption on non-binge days was comparable to individuals with obesity without BED. Along the same line, HC-OW and HC-OB did not choose more high-calorie foods, nor did they like them better. This contradicts the common preconception that individuals with higher body weight generally make poor food choices (see e.g., 43 for media representations of obesity) or follow unhealthy food preferences. Our results question this simplistic view of overweight and obesity that contributes to weight stigma [44].

Some previous work has reported increased impulsivity in BN and BED [45–47], which could have been reflected in faster and more frequent choices for higher-calorie foods. However, this was not the case in food choice outcomes in our study. The only result that could possibly reflect increased choice impulsivity was observed in decision times. Both individuals with BN and AN, as well as individuals with HC-OW/OB were generally faster in choosing foods than HC-NW, possibly pointing towards lower choice conflict [18] or more automatic choices in the sense of dual process-models (see e.g., [16]). This was not the case for individuals with BED. The results are in line with previous research finding increased impulsivity in BN [47] but no evidence for rapid response impulsivity in BED [48]. However, notably, these faster choices were not related to higher-calorie choice outcomes in any of the groups. Previous studies using reaction time measures found an approach bias for foods (i.e., faster reactions for approaching foods than objects) in an analogue sample with subclinical BN [49] but not in a sample of diagnosed BN [50]. Matching our results that BED did not make faster food choices (compared to HC) previous research found no approach bias for high-calorie foods in BED [51]. The evidence for faster food-related choices or approach biases is therefore mixed. Future studies might investigate the underlying process of these groups making faster choices without this leading to increased choices of higher-calorie foods. Joint analyses of food choices and decision times may further differentiate choice processes by illuminating the cognitive processes underlying food choices (for an example in BN see 20).

Given that the different groups, apart from AN, were surprisingly similar in the number of chosen calories, we wanted to know which other characteristics might explain food choice in the large cluster comprising normal weight, overweight, obesity, BN, and BED. We expected that BMI or eating styles might be strong predictors of food choice (see [17, 52]). Of the eating styles emotional and external eating, measured by the DEBQ, only the latter significantly related to food choices: those scoring higher on external eating chose more calories. External eating can be seen as a reflection of reactivity to food cues: seeing a food and choosing it instead of attending to internal cues. Food cue reactivity has been linked to weight gain in experimental and prospective studies [53]. The lack of an effect of emotional eating style may also be due to low negative affect during the experiment. Future studies might reinvestigate food choice in an emotionally negative condition. However, note that Gianini et al. [19] did not find an effect of negative emotion on food choice task outcomes in BN. Surprisingly, BMI had no relationship with food choice. This might partly be due to the opposing pattern of BMI effects in HC and BED [17]: We had previously seen that BMI is associated negatively with food choice in HC but not in individuals with BED. Also, the severity of eating disorder symptoms, measured by the EDE-Q did not affect choice.

Regarding transdiagnostic integration across groups, a lot is known about eating outcomes in the different groups, i.e., *what* they eat (strong restriction, favouring lower-calorie foods in the case of AN vs. episodes of binge-eating high-calorie foods in BN/BED). However, we know less about the *how* of these outcomes, i.e., what processes lead to these outcomes? Food decision-making studies are one way to investigate these processes. Our study showed that the ‘how’ of food choice in both AN and BN is ‘faster’. More studies are needed to investigate whether this choice speed is a reflection of the same process across groups or whether it reflects different processes. It is conceivable that in AN it may reflect strong food choice habits (see e.g., [54, 55]) as a result of their food avoidance whereas in BN it may reflect impulsivity (see e.g., [47, 56]). Further research is needed about ‘what’ the circumstances leading to the outcomes are. Factors might include general impulsivity [57], food avoidance/fear [58], emotional states [35] or contextual factors [59]. Our results further showed that the binge-episodes characterizing BN and BED are not reflected in our food choice outcome. A differentiation of their eating episodes during and between binges may be helpful. Future studies might use ecological momentary assessment to track eating episodes in daily life. Within the laboratory, having participants verbalise their thoughts during food choice [60] might yield insights beyond quantitative results. Individualisation of food stimuli to personal binge-foods/preferences might also change outcomes.

As we decided to use a food choice task to investigate food-specific processing in eating disorders and weight groups, our results do not exactly map the dimensions of the Research Domain Criteria Framework (RDoC; [61]) that is often used for transdiagnostic research. Nevertheless, our results do have some overlap with RDoC dimensions. Within the Positive Valence Systems domain the task taps into reward valuation in eating disorders [62] and may indicate blunted food reward in AN (decreased liking). However, no increased reward expectancy was observed in the other groups (no difference from HC in food liking). Within the Reward Learning subdomain of habit, AN’s fast and consistent lower-calorie choices may point to a learned habit [55], while no opposite pattern was observed in the groups with binge eating. While choice tasks might also be used to investigate the RDoC subdomain of response inhibition, we cannot directly infer something about response inhibition from our task design. Future studies might include instructions more tailored to evoke response inhibition i.e., to ask AN to always choose the higher-caloric option.

### Limitations and future directions

Our study has the strength of comparing several groups with diagnosed eating disorders and of different weight to explore food choice processes. However, our approach also has some limitations. First, the groups with AN and BN were in in-patient treatment when they participated in the study. This means that their study protocol differed slightly: standardising their lunch was not possible because they followed their treatment eating plans. Second, while the food-choice task included a broad range of different foods, food choices were always binary. To make choices more realistic, future studies could include multiple options [63], the possibility to not choose any of the options or self-selected meals [64]. Third, although we were able to recruit a considerable number of individuals with eating disorders for our study, sample sizes of the groups were limited. Fourth, decision time can be a reflection of different processes, e.g., impulsivity, i.e., rapid decisions disregarding consequences [65], habit, i.e., situationally bound, rather automatic decisions [66] or choice difficulty, i.e., the result of weighing different aspects of the choice [67]. Disentangling these would either require a full choice path analysis [68] or brain imaging methods. Consequently, further studies are needed to clarify which processes underlie decision times in the different groups. Fifth, we did not control for any additional biological factors that could affect food choice such as menstrual cycle stage (see e.g., [69]), specific deficiency symptoms or energy expenditure.

An interesting direction for future studies is longitudinal developments of choice process measures. Looking at choice outcomes cross-sectionally, individuals with AN later in treatment chose more high-calorie foods [18]; longitudinally, increases in selecting high-fat foods and in choosing tasty rather than healthy foods were related to healthier weight trajectories after discharge from the hospital [23]. For choice-process measures (e.g., decision times or mouse-tracking) as a measure of conflicted choice little is known about their longitudinal development (for cross-sectional differences in mouse-tracking measures see 18) and how it is associated with behavioural outcomes.

The results of this study concerning calorie choice partly differed from primary analyses of the data [15, 17, 18] which found individuals with BN and BED to choose high-calorie foods more often than HC. This stems from differences in samples and analytic approaches: In both previous papers, control participants were matched on BMI and age to resemble individuals with BN [18] and BED [17]. For this paper, this was not possible because BN and BED differ both in average BMI [70] and eating-disorder onset [71]. Instead, all participants with sufficient trial numbers were included in the analysis. The sample was therefore not identical to previous papers. Regarding statistical analysis, the previous papers modelled choice outcomes based on the calorie difference between the two foods based on individual estimates of calorie content. In this paper, objective calorie content of the chosen food was analysed instead. The previous approach therefore focused on how the *difference* in *subjective* calorie content influenced the *probability* of choice; the present account focused purely on *objective* choice *outcome.* This is an important methodological aspect to be considered in the design and analysis of future food choice studies.

## Conclusions

The study showed that AN was the only group clearly distinct from HC-NW in their food preferences and liking. Importantly, all other groups showed similar choice patterns to HC-NW. This challenges the common stereotype that associates overweight, obesity and binge eating with poor food decision-making. The findings emphasise the importance of moving beyond calorie choice outcomes to investigate the underlying cognitive and emotional processes driving food decisions. Incorporating measures of decision-making speed, emotional states, and contextual factors into future research could provide a more comprehensive understanding of eating behaviours across diagnostic and weight groups.

## Supplementary Information

Supplementary Material 1.

## Data Availability

The datasets supporting the conclusions of this article are available in the Psycharchives repository 10.23668/psycharchives.21660.

## Abbreviations

AN: Anorexia nervosa
BN: Bulimia nervosa
BED: Binge-eating disorder
HC: Healthy controls
HC-NW: Healthy controls with normal weight
HC-OW: Healthy controls with overweight
HC-OB: Healthy controls with obesity
BMI: Body mass index
DEBQ: Dutch eating behaviour questionnaire
EDE-Q: Eating disorder examination questionnaire
RdoC: Research domain criteria framework

## Footnotes

1 A German version of the interview was not available at the time of testing. Diagnosis changes from DSM-IV to DSM-V were coded manually.

2 We have no information about the treatment status of the group with BED.

3 cheese, burger, peanuts, muffin, pizza, chocolate-coated marshmallows, pizza, chocolate, crisps, sausages.

4 apple, banana, mushrooms, gherkin, carrot, crispbread with cottage cheese, kohlrabi, lettuce, tomatoes.
