# Supplementary material for: Food preference and behavioural choice across the eating disorder and body weight spectrum
Source: J Eat Disord. 2026 Feb 14;14:66. doi: 10.1186/s40337-026-01547-4 (PMC13011405; doi:10.1186/s40337-026-01547-4)
Supplement: Supplementary file 1 — Supplementary Material 1. [file 40337_2026_1547_MOESM1_ESM.docx]

**Supplement**

**Images used for food choice task**

The following pictures from the food-pics database (Blechert et al., 2019) have been used for the food choice task: apple (192), banana (282), mushrooms (brown) (263), gherkin (380), carrots (431), crisp bread with cottage cheese (225), kohlrabi (268), lettuce (228), tomatoes (197), camembert (519), cheese burger (65), peanuts (183), chocolate muffin (80), pizza (salami) (61), mini chocolate marshmallow (161), bar of chocolate (286), chips (117), sausages (535).

**Group-specific correlations between healthiness and liking**

We also explored the correlations of perceived healthiness and liking between groups. The two dimensions were uncorrelated in BED (*r* (22) =-.01) and BN (*r* (27) = .04). In all groups without eating disorders, there were small positive correlations between healthiness and liking (HC-NW: *r*(55) = .10; HC-OW: *r* (16) = .19; HC-OB: *r* (19= .21). Only in AN was the correlation strong (*r* (38) = .50). Please note that healthiness and calories were highly correlated for this sample and set of stimuli (*r*(188) =. 90).

**Sample and data overlap**

The data used for this manuscript comes from a larger project (‘NewEat’) for which participants completed several different tasks. As such, other papers have been published previously which have a partial sample overlap with the sample analysed for this manuscript. In addition, this paper partially reanalyses published decision-making data to allow for an extensive group comparison. For an overview, see below.

The reanalysed data from the food choice task was used in the following three papers: one paper on restrained eating in individuals without eating disorders (DOI: 10.1007/s00426-019-01185-3), one with a focus on food choice and choice processes in in anorexia nervosa and bulimia nervosa (DOI: 10.1016/j.appet.2021.105745) and one with a focus on food choice and choice processes in binge eating disorder (DOI: 10.1016/j.appet.2021.105890).

In addition, the following papers have an overlap in samples but concern data from other tasks: Emotional food cue-reactivity in BED (10.1002/eat.23683), emotional eating in individuals without eating disorder (10.1007/s40519-023-01606-8), emotional eating across different eating disorders ( 10.1002/eat.23477), emotional food cue-reactivity in AN and BN (10.1002/erv.2849 and 10.1002/eat.24028), emotional food cue-reactivity in individuals without ED (10.3389/fnbeh.2020.00091), emotional food craving (10.31234/osf.io/2acnb), emotional eating questionnaires (10.1080/10640266.2019.1642036) and interoceptive sensitivity (10.1002/erv.2676).

Blechert, J., Lender, A., Polk, S., Busch, N. A., & Ohla, K. (2019). Food-Pics_Extended—An Image Database for Experimental Research on Eating and Appetite: Additional Images, Normative Ratings and an Updated Review. *Frontiers in Psychology*, *10*. https://doi.org/10.3389/fpsyg.2019.00307
